# Diagnosis of *Chlamydia trachomatis* genital infections in the era of genomic medicine

**DOI:** 10.1007/s42770-021-00533-z

**Published:** 2021-06-23

**Authors:** Seema Shetty, Christina Kouskouti, Uwe Schoen, Nikolaos Evangelatos, Shashidhar Vishwanath, Kapaettu Satyamoorthy, Franz Kainer, Angela Brand

**Affiliations:** 1 Department of Microbiology, Kasturba Medical College, Manipal, Manipal Academy of Higher Education, Madhav Nagar, Manipal, 576104 Karnataka India; 2 United Nations University – Maastricht Economics and Social Research Institute On Innovation and Technology (UNU-MERIT), Maastricht, 6211 AX The Netherlands; 3 Manipal Centre for Infectious Diseases, Prasanna School of Public Health, Manipal Academy of Higher Education, Manipal, 576104 Karnataka India; 4 Department of Obstetrics and Perinatal Medicine, Klinik Hallerwiese, St. Johannis-Muhlgasse 19, 90419 Nuremberg, Germany; 5 Division of Maternal and Fetal Medicine Department of Obstetrics and Gynaecology, Mt. Sinai Hospital University of Toronto, Toronto, ON, Canada; 6 BioMedHeliX (Pty) Ltd., 3 Conifer Road, Cape Town, 8005 South Africa; 7 Interdepartmental Division of Critical Care Medicine, University of Toronto, ON, Canada; 8 Dr. TMA Pai Endowment Chair in Research Policy in Biomedical Sciences and Public Health, Prasanna School of Public Health (PSPH), Manipal Academy of Higher Education, Manipal, 576104 Karnataka India; 9 Department of Cell and Molecular Biology, Manipal School of Life Sciences, Manipal Academy of Higher Education, Manipal, 576104 Karnataka India; 10 Dr. TMA Pai Endowment Chair in Public Health Genomics, Department of Public Health Genomics, Manipal School of Life Sciences, Manipal Academy of Higher Education, Manipal, 576104 Karnataka India; 11 Department of International Health, Faculty of Health, Medicine and Life Sciences, Maastricht University, Maastricht, 6229 GT The Netherlands

**Keywords:** *Chlamydia trachomatis*, Genital infections, Diagnostics, NAAT, Point of care, -omics’

## Abstract

**Purpose:**

Chlamydial genital infections constitute significant sexually transmitted infections worldwide. The often asymptomatic status of *C. trachomatis* (CT) infections leads to an increased burden on human reproductive health, especially in middle- and low-income settings. Early detection and management of these infections could play a decisive role in controlling this public health burden. The objective of this review is to provide an insight into the evolution of diagnostic methods for CT infections through the development of new molecular technologies, emphasizing on -omics’ technologies and their significance as diagnostic tools both for effective patient management and control of disease transmission.

**Methods:**

Narrative review of the diagnostic methodologies of CT infections and the impact of the introduction of -omics’ technologies on their diagnosis by review of the literature.

**Results:**

Various methodologies are discussed with respect to working principles, required specifications, advantages, and disadvantages. Implementing the most accurate methods in diagnosis is highlighted as the cornerstone in managing CT infections.

**Conclusion:**

Diagnostics based on -omics’ technologies are considered to be the most pertinent modalities in CT testing when compared to other available methods. There is a need to modify these effective and accurate diagnostic tools in order to render them more available and feasible in all settings, especially aiming on turning them to rapid point-of-care tests for effective patient management and disease control.

## Introduction

*Chlamydia trachomatis* (CT) infections impose a huge burden on human sexual and reproductive health. They are considered to be the most significant causes of bacterial sexually transmitted infections (STIs) worldwide [1–3]. The World Health Organization (WHO) reports 357 million new cases of four major STIs each year: chlamydia, gonorrhea, syphilis, and trichomoniasis. Among these, chlamydia infections make a substantial contribution of about 131 million cases [4]. According to reports by the Centers for Disease Control and Prevention (CDC), chlamydia infections are the most frequent notifiable diseases in the USA and constitute a major proportion of all STIs reported to CDC [5]. As CT is mostly an asymptomatic pathogen and may not often cause classical clinical features, many cases remain undetected, which leads to an underestimated prevalence rate. This fact highlights the paramount importance of using effective diagnostic modalities in providing better patient care. To provide an insight into the various diagnostic tools utilized for effective patient management and control of disease transmission, we present a literature review of the diagnostic methodologies of CT infections and the impact of the introduction of -omics’ technologies on their diagnosis.

### The pathogen

Chlamydia is an obligate intracellular pathogen characterized by a distinctive life cycle involving dual forms: an infectious extracellular elementary body (EB) and an intracellular reticulate body (RB) for replication. These bacteria grow only within the specialized vacuoles called inclusions in eukaryotic cells. CT comprise the most popular species in the family *Chlamydiaceae*, because of its association with ocular trachoma and a wide array of genital tract manifestations in both males and females [6]. Originally, CT isolates were classified by serotyping, based on antigenic alterations on the major outer membrane protein (MOMP), and linked to different clinical manifestations [7, 8]. To date, 19 serovars of CT (A, B/Ba, C, D/Da, E, F, G/Ga, H, I/Ia, J, K, L1, L2/L2a, and L3) have been described. However, serotyping requires the cultivation of CT on cell lines, which deems it cumbersome and insensitive. Nowadays, molecular typing methods can be used for genotyping, involving analysis of the *ompA* gene encoding for MOMP; thus the cultivation of cell lines is no longer required. The various genovars were named using similar letter-based nomenclatures as for serovars. Some of the typing methods involved are ompA sequencing, multilocus sequence typing (MLST), multilocus variable number tandem repeat analysis (MLVA), and whole-genome sequencing (WGS). In addition to the 19 serovars mentioned above, genovars Ja and L2b have been recognized [9, 10]. The various genovars reported in studies across the globe have been well summarized by Rawre J et al., with genovar E emerging as being the most frequent [9].

### Clinical presentation

Several risk factors are reported that may contribute to developing CT infections. The sexually active younger age group of < 25 years is considered to be at a higher risk of acquiring the infection. Other major factors include multiple sex partners, inconsistent usage of barrier methods with new sexual partners, past history of STIs, and exposure to commercial sex partners [8, 11]. Genital infections caused by CT are mostly asymptomatic in 70–80% of women and 40–50% of men, leading to a huge reservoir of undetected, infected individuals who pose a major threat of transmitting infections to their sexual partners [12]. In males, CT infections most commonly present as non-gonococcal urethritis (NGU), with other common presentations including epididymitis and proctitis. Invasive serovars (L1-L3) linked with lymphogranuloma venereum (LGV) are mostly reported in men who have sex with men (MSM) and present with infections involving the inguinal lymph nodes and rectum [8, 13]. In females, the most common clinical presentation of a CT infection, when symptomatic, is mucopurulent cervicitis, which may be associated with urethritis. However, in the majority of women, CT infections run their course asymptomatic and these undetected and untreated cases may lead to sequelae of clinical complications influencing reproductive health. When an unidentified CT infection spreads to the upper genital tract, it can cause pelvic inflammatory disease (PID) including a varied range of manifestations such as endometritis, salpingitis, tubo-ovarian abscesses, pelvic peritonitis, and perihepatitis. Chronic PID can further lead to more severe complications such as tubal factor infertility and ectopic pregnancy [14]. Furthermore, an increased risk of acquiring cervical carcinoma and infection with human immunodeficiency virus (HIV) has been found to be related to active chlamydial infection. In both genders, accidental autoinoculation of genital secretions may lead to inclusion conjunctivitis [12, 15].

During pregnancy, CT infections have been mostly associated with adverse outcomes such as recurrent miscarriages, stillbirth, premature rupture of membranes, preterm labor with low birth weight, and postpartum endometritis. Maternal infection can be transmitted to the neonate during vaginal delivery, through contact with infected cervical secretions, potentially leading to neonatal inclusion conjunctivitis, otitis media, and infant pneumonia. Long-term respiratory sequelae may be significant in these children when followed-up [16, 17].

Being a silent pathogen, most of the infections remain concealed and may later be revealed to have influenced the reproductive health, causing female sterility, which in turn brings with it an associated financial burden. Adequate screening programs need to be implemented in order to diagnose such infections. CDC recommends annual screening for chlamydia among sexually active females aged < 26 years and in older females associated with risk factors, although there is no emphasis on routine screening in men [1, 15]. However, as these screening programs are rarely being implemented, especially in developing and underdeveloped countries, the number of new cases every year keeps increasing [18].

From all the above, it becomes clear that the real challenge in managing CT infections is to identify these silent infections. This requires efficient diagnostic methods with adequate sensitivity and specificity, which, when implemented as part of screening programs, can contribute to detecting new cases and controlling the transmission of infections. This review focusses on the currently available diagnostic approaches in detecting chlamydial infections, with special emphasis on the significance of -omics’ technologies in molecular testing.

### Clinical specimens in CT testing

CT diagnosis involves various direct and indirect laboratory-based methods whose efficiency depends primarily on the adequate collection and transportation of clinical specimens. As chlamydia is an obligate intracellular bacteria, specimens collected must involve host cells harboring the pathogen, especially when direct methods are involved in testing [19]. Also, the type of suitable specimens could vary based on the clinical presentations and the testing method [20].

Genital swabs are known to be the most commonly collected specimens for the detection of chlamydial infections. Dacron, rayon, cotton, and calcium-alginate tipped swabs are generally preferred [19, 21]. In women, cervical swabs or cervical swabs pooled with urethral swabs and in men urethral swabs are considered to be effective for chlamydial isolation. Present studies emphasize the collection of non-invasive specimens for testing like self-collected vaginal swabs in females and first-void urine in both males and females [22, 23]. The testing for extra-genital CT infections requires sampling from rectal and pharyngeal sites [24].

Genitourinary specimens collected from various anatomical sites can influence diagnosis owing to the difference in their bacterial load. Michel et al. have elaborated on the importance of bacterial load in varied specimen types and their association with clinical symptoms [25]. In men, urethral swabs and first-void urine specimens are considered equally adequate as there is no significant variation in terms of bacterial load. In females, though endocervical swabs are the preferred specimens, self-collected vaginal swabs have proved to have comparable significance with almost similar bacterial load. However, first-void urine in females is considered sub-optimal because of the poor bacterial load [25, 26]. Higher chlamydial load is found to correlate with symptomatic presentation in both males and females and also considered as an important indicator for transmission and development of sequelae [27].

## Traditional methods in CT testing

### Cervical cytology

Cervical cytological examination using Papanicolaou (Pap) smears has been among the early techniques adopted in screening for cervical cancer. This simple, rapid, readily accessible method is also known to provide clues in diagnosing STIs, with the associated inflammatory changes [28]. Several studies have been conducted to assess the utility of this method in CT diagnosis in the past [29–34]. Some studies have highlighted the association of cervical epithelial changes and cytological variations including increased histiocytes and transformed lymphocytes with CT infections, indicating Pap smears as screening tests [28, 29]. However, these changes may not be unique to CT infections [30]. In addition, ample data on poor sensitivity of this methodology, provided by various research reports, has marked it as an unreliable method both in screening and diagnosis of CT infections [31–34].

### Culture methods

Isolating CT on cell cultures is considered to be the most optimal method of detecting urogenital infections caused by the organism. Because of the high specificity of this technique, it had been referred to as the “gold standard” for CT detection in the past. These cell culture methods require invasive specimens like endocervical and urethral swabs which must be collected precisely to include host cells with viable organisms. They must be transferred in suitable transport media like sucrose-glutamate phosphate buffer and transported at a temperature < 4 °C within 24 h of collection. If the processing within 24 h is not secured, transport at a temperature of − 70 °C on dry ice is required [19]. Cell lines mostly utilized include McCoy, Buffalo green monkey kidney cells and HeLa 229. These are analyzed for the presence of characteristic intracytoplasmic inclusion bodies at least 48–72 h after inoculation [7]. However, stringent conditions required in collection, transport, and processing of specimens pose a major drawback influencing the sensitivity, which may range from 70–80%, sometimes being even lower. These methods are technically intricate, labor-intensive with increased turnaround time and cost. Hence, these are no more preferred in routine diagnostic setups but are still important in studying drug resistance and in situations with legal implications as in sexual abuse [35, 36].

### Antigen detection methods

Due to these drawbacks of culture methods in CT diagnosis, various other methods, which are non-culture based, have been developed and implemented. Methods demonstrating CT antigens from clinical specimens are among these improved techniques. As these methods do not require viable bacteria, highly specific procedures involved in specimen collection and transport, as needed for culture samples, are not necessary. Also, the advent of these methods has made CT testing possible in laboratories lacking the capability to maintain cell cultures.

Direct fluorescence antibody (DFA) test performed directly on clinical specimens is one of the most useful diagnostic methods available. This uses fluorescein isothiocyanate-labeled monoclonal antibodies directed against chlamydial lipopolysaccharide (LPS) or MOMP of CT to detect chlamydial inclusions. Using monoclonal antibody reagents specific for MOMP of CT has led to 98–99% specificity and 80–90% sensitivity in comparison with cell culture methods. Very high specificity shown by the DFA test owes to it being dependent on visualizing distinctive morphology and staining properties of chlamydial inclusions as in cultured cells. This test is relatively rapid and can be performed in about 30 min. It does not depend on a temperature-controlled transportation system. But this technique demands expertise and time in examination and interpretation of results, thus confining its use in low volume setups. However, due to its high specificity, it has been used as a confirmatory test for positive results obtained by other non-culture tests [12, 19, 21].

Enzyme immunoassays (EIAs) based on the detection of LPS genus-specific antigen was introduced later in diagnostics. These tests are based on LPS which is more abundant and soluble than MOMP. The major drawback of these techniques is the cross-reaction of LPS-specific antibodies to LPS of other gram-negative bacteria, leading to false-positive results. In order to improve specificity, certain blocking assays were developed which consist of re-testing the previously positive results in the presence of monoclonal antibodies specific to chlamydial LPS. As a result, these confirmatory tests increased the specificity of EIAs from 97 to > 99% rendering them as appropriate screening tests, even in a population with low prevalence [13, 19]. The efficiency of Chlamydiazyme (Abbott Diagnostics, North Chicago, Ill.) and MicroTrak (Behring, California, USA) assays have been analyzed by various studies in comparison to culture and other non-culture methods such as DFA, nucleic acid hybridization (NAH), and nucleic acid amplification (NAA) [37–43]. The sensitivity was determined to be 65–75% with a specificity of 97–99%.

### Serology testing for CT

The role of serological techniques in CT diagnosis is very limited, as they have been proven to be inadequate. The chlamydial antibody response is either delayed or absent in some individuals. Most of these techniques detect anti-chlamydial antibodies directed against LPS antigen which is a genus-specific antigen. Often these tests fail to differentiate antibodies produced against different species of chlamydia. As a result, these tests are not recommended as part of screening programs. However, they may be of diagnostic importance in chronic and invasive infections like in cases of PID and sexually acquired reactive arthritis (SARA) where direct detection of CT from genital specimens is not possible but found to be associated with increased anti-chlamydial antibody titers [7, 8].

Some of the serological tests evaluated for the diagnosis of chlamydial infections are the microimmunofluorescence (MIF) test and enzyme-linked immunosorbent assay (ELISA). The MIF test was believed to be the most sensitive serological test, which even presented species and serovar-specific responses. This was regarded as the “serological gold standard” for chlamydial testing [44]. However it has not been established for diagnostics because of technical constraints and cost involved. ELISA tests using genus-specific LPS are known to produce false-positive reactions attributable to cross-reactions with other chlamydial species. As antibodies produced during previous infections generally remain in circulation, positive test results do not confirm the presence of an active infection. The presence of IgM antibodies as expected in acute infections is also not consistent and a single serum specimen tested by ELISA fails to distinguish between previous and present infections. However, testing serum for chlamydial antibodies can be indicative of past infections and related to tubal infertility [45].

## Modern technologies in CT diagnosis

### Molecular techniques

Traditional methods for the diagnosis of CT infections have several disadvantages including low sensitivity, the requirement of invasive specimens, longer duration for performance and reporting, and associated high cost. Also, these tests may produce false-negative results, which can in turn lead to the spread of infections and increase in complications. These limitations point out the need to develop tests with higher sensitivity and specificity, in order to be used either as standard standalone reference tools or in combination with other traditional methods. The introduction of these methods led to a drastic leap in CT diagnosis, enabling better identification of cases and management.

Commercially available nucleic acid hybridization (NAH) methods have been used in laboratories with high specimen load. The most commonly used method is the PACE 2 test (Gen-Probe, San Diego, Calif., USA). This method uses a chemiluminescent DNA probe that hybridizes to a species-specific sequence of chlamydial 16S rRNA. Another DNA probe test developed in the later years is the PACE 2C which simultaneously detects both CT and *Neisseria gonorrhoeae* from a single specimen [12]. One other NAH test being used is the Hybrid Capture II (Digene Corporation, USA) using a signal amplification component so that the sensitivity can be increased [13]. These relatively rapid, simple tests have been suitable for testing a large number of specimens. Data on the performance of these NAHs show that they have almost comparable sensitivity and specificity to that of other EIAs [40, 46, 47]. In comparison to DNA amplification assays, they are found to have decreased sensitivity [48]. Special care is necessary when confirming positive results especially from populations with low prevalence.

The development of nucleic acid amplification tests (NAAT) has been regarded as a major breakthrough in CT diagnostics and has utterly transformed the field. These tests can detect chlamydial DNA or RNA based on amplification technology and have almost succeeded in replacing cell culture methods, being referred to as the “new expanded gold standard.” These tests demonstrate high sensitivity, as they are efficient enough to detect the presence of a single nucleic sequence in the clinical specimen and are at the same time highly specific. Additionally, these tests have introduced the concept of non-invasive sampling for diagnosing genital CT. Studies have shown their efficacy in detecting CT in self-collected vaginal swabs and urine specimens [22, 23, 49, 50]. As the majority of these infections is asymptomatic, the collection of non-invasive specimens has proven beneficial for screening programs. First-void urine is the recommended specimen in men, while in women, self-collected or clinician-collected vaginal swabs are preferred for NAAT testing [51].

The major role of NAAT technologies in chlamydial diagnostics was appreciated with the development of polymerase chain reaction (PCR) and ligase chain reaction (LCR) techniques. Both of these methods involve the amplification of nucleotide sequences within a cryptic plasmid [52]. In 1993 the first PCR test approved by the Food and Drug Administration (FDA) was Amplicor (Roche Molecular Systems, Incorporated, Branchburg, NJ), which was approved for endocervical, male urethral and male urine specimens. Moreover, some studies have shown equally good sensitivity of this method, when tested on female urine samples [53]. Furthermore, it showed higher sensitivity in comparison to culture methods [54] as well as to non-culture techniques [42]. Studies have even highlighted the utility of this test in detecting CT from extra-genital sites, thus being useful in screening MSM [55]. However, the presence of inhibitors in clinical specimens and the poor handling of specimens have been found to alter the performance of this PCR technique. In 1995, LCR received approval by the FDA for diagnostic use. LCx assay (Abbott Laboratories, Abbott Park, Ill), an oligonucleotide probe-based assay, uses two probes that are ligated together when adjacent to each other and hybridize to one strand of the target DNA [56]. The evaluation of the performance of this method exhibited good sensitivity and specificity in comparison to the cell culture method, making it a useful tool for screening urogenital infections [57, 58]. PCR and LCR demonstrate almost similar results when used in the diagnosis of CT and are considered as standards in testing and confirmation [59].

Following PCR and LCR, newer molecular technologies have been developed for CT diagnosis, which include the transcription-mediated amplification (TMA) test, the APTIMA Combo 2 (Gen-Probe Inc, USA), and the strand displacement amplification-based ProbeTec (BD Diagnostic Systems, USA). The TMA test is directed against ribosomal RNA, which works as an isothermal system using enzymatic target amplification and chemiluminescent detection in a single tube format [19]. FDA has cleared this technique in 2005 for testing genital and urine specimens [60]. Gaydos CA et al. have reported a sensitivity of 94.2 and 94.7% and a specificity of 97.6 and 98.9% when testing endocervical swabs and first catch urine respectively with this method [61]. In a comparative study conducted by Lowe P et al., the APTIMA Combo 2 assay showed very high sensitivity (100%) and specificity (> 99%) compared with the Amplicor CT/NG assay [62]. The ProbeTec assay, targeting the chlamydial cryptic plasmid, was another significant development for CT diagnosis and was approved by the FDA in 2014. Sensitivity was almost comparable to the other NAATs, while it showed a specificity of 100% as demonstrated by Van Dyck E et al. [42].

Another FDA cleared NAAT is the Cepheid Xpert CT/NG (Sunnyvale, CA), a rapid, easy-to-use, cartridge-based real-time PCR. This assay has been proven to be highly effective in testing urogenital specimens from both symptomatic and asymptomatic population [63]. Equally good performance was demonstrated when testing extra-genital specimens [60]. FDA has approved Aptima Combo 2 assay and Xpert CT/NG for extra-genital diagnostic testing of chlamydial infections using pharyngeal and rectal specimens, in 2019 [64]. Xpert CT/NG is considered to be the first molecular method to be utilized as a point-of-care (POC) assay. Being a closed system requiring minimal manual intervention, it is easy and rapid to perform. These properties allowed for it to be used even in clinics lacking conventional laboratory facilities, thus enabling same-day treatment to patients. This has contributed to preventing loss of cases to follow up and occurrence of severe sequelae [65].

NAATs have been the most competent methods devised for CT diagnosis with high sensitivity and specificity values. The fact that non-invasive samples are accepted for testing has favored the use of these methodologies in screening asymptomatic population. These are found to be considerably rapid, while providing accurate results. They are even considered efficient enough to replace culture methods in testing specimens implicated with legal issues. Most of these test formats are designed to detect both CT and *N. gonorrhoeae* in the same assay using a single specimen and therefore enabling better patient management. However, the requirement of well-trained personnel for testing specimens and the necessity of sophisticated laboratory infrastructure for most of these assays have limited their usage to high-end settings. Additionally, the high financial cost of these methods has prevented their popularity in resource-poor setups.

### “Swedish variation” of CT

Though a highly conserved pathogen, genetic variation involving a 377 base pair deletion in the cryptic plasmid has been noted for CT. This variant denoted as nvCT was first reported from Sweden in 2006 by Ripa and Nilsson and has been associated with reduced transmission duration. Earlier versions of some commercial NAAT systems had failed to detect this variant as the targets for these NAATs were located in the acquired deletion site of the plasmid. While designing in-house PCRs or using commercial NAAT systems for CT detection, ability to detect this variant should be considered to avoid false-negative results [66–68].

## Rapid “Point-of-care” testing in CT diagnosis

“Point-of-care” testing (POCT) in a public health setting provides major advantages compared with the often cumbersome sample logistics of medical laboratories. The relatively low costs of the testing equipment allow more installations closer to the patient. This also leads to a faster availability of the results for the patient and medical care team and, in eligible cases, an immediate commence of treatment. The so achieved initiation of treatment during the first visit to the health facility could result in higher rates of follow-up.

The availability of POCT in clinical settings is considered essential in controlling chlamydial infections. According to WHO, such tests should be affordable, sensitive, specific, user-friendly, rapid and robust, equipment-free, and deliverable to end-users (ASSURED criteria) [69]. Chlamydial diagnosis involves highly accurate NAATs. However, these have been only accessible in the developed world, as they demand well-equipped laboratories with trained personnel and high maintenance costs. As these tests are mostly laboratory-based, the patients are required to make a second visit to the clinic for collecting test results and receiving instructions for treatment. Patients in middle- and low-income countries may fail to access these technologies, leading to an increased burden of infections. Rapid and accurate POC tests are necessary in order to enable diagnosis and treatment on a single clinical visit. This would decrease the rate of patients’ lost-to-follow-up, further reducing morbidity and potential transmission [70].

Immunochromatography-based rapid diagnostic tests (RDTs) detecting chlamydial LPS antigen have been developed, which require minimal logistics and deliver rapid results enabling treatment of positive cases immediately. But poor sensitivity made them inappropriate for testing both symptomatic and asymptomatic patients [71–73]. To overcome this weakness, molecular RDTs were introduced in POCT. The Cepheid GeneXpert platform is an example for an established POCT system which even works well in mobile laboratories. Xpert CT/NG can provide highly accurate results, comparable with laboratory-based NAATs, in approximately 90 min, thus facilitating faster delivery of results and immediate administration of treatment [74]. Furthermore, the method has greatly benefitted from the FDA approval of testing extra-genital specimens. Harding-Esch EM et al. have also presented a NAAT-based RDT, which can produce results in 30 min with a performance analogous to the standard reference tests [75].

The sensitivity, specificity, advantages, and disadvantages of various diagnostic tests available for CT detection are summarized in Table 1.

**Table 1:** Comparison of diagnostics in detecting genital infections by *C. trachomatis*

Test method	Sensitivity (%)	Specificity (%)	Advantages	Disadvantages	References
Cell culture	70–85	99.9	• Detection of viable bacteria • Availability of bacteria for genotyping and antimicrobial susceptibility testing	• Stringent collection and transport of specimens • Technically complex • Time-consuming • High cost	Domeika et al. [8] Black CM [19]
Antigen detection Methods					
a) Direct Fluorescent Antibody test	50–90	98–99	• Visualizing morphology of inclusions • Assessment of quality of specimens	• Expertise in interpretation • Not suitable for large sample size	Black CM [19] Gann et al. [41] Phillips et al. [76] Dereli et al. [77]
b) Enzyme immunoassay	65—80	97–99	• Minimal technical skills	• False-positive reactions • Time-consuming	Chernesky [13] Clarke et al. [40] Gann et al. [41]
c) Immunochromatography	20–65	97–99	• Rapid • Easy to perform	• Poor sensitivity	Kelly et al. [69] Sabido et al. [71] Yin et al. [72]
Molecular methods					
a) NAH	75–85	97–99	• Relatively simple, rapid • Suitable for large specimen numbers	• The requirement of specific instruments • Low sensitivity	Clarke et al. [40] LeBar et al. [47] Black et al. [48]
b) NAAT	84–99	92–99	• High sensitivity • Rapid, accurate • Use of non-invasive specimens	• Expensive • Sophisticated laboratory infrastructure • Well trained personnel	Black et al. [48] Harkins et al. [52] Gaydos et al. [61]
c) CBNAAT	97–100	97–100	• Cartridge-based • Near point-of-care test • Testing extra-genital specimens	• High cost	Gaydos et al. [63] Garrett et al. [78]

*NAH* nucleic acid hybridization, *NAAT* nucleic acid amplification test, *CBNAAT* cartridge-based nucleic acid amplification test

## CT diagnosis in the era of -omics’ technologies

The advent of -omics’ technologies has led to a major revolution in diagnostics of a wide array of clinical conditions. These assays can prove to be instrumental in managing various infectious diseases, as they could reveal critical details of the pathophysiological mechanisms involved in health and disease. These methods in combination with bioinformatics have contributed immensely to understanding the pathophysiology and complex interplay between hosts and pathogens. The wider term “-omics” encompasses a range of technologies for analyzing and detecting genes (genomics), messenger RNA (mRNA) (transcriptomics), proteins (proteomics), and metabolites (metabolomics) from clinical specimens, which could prove to be important biomarkers in various clinical conditions. These technologies, especially when utilized in an integrated manner, could evolve as powerful diagnostic tools in clinical medicine [79, 80].

The utility of -omics’ in CT diagnosis has evolved over the years. Despite its high infection rates and impact on human health, the unique biology of these obligate intracellular bacteria has not been well explored until the introduction of -omics’ investigations. These modern modalities have been fundamental in shedding light on the natural history, evolutionary aspects, and complex pathophysiology of this mysterious pathogen, thus paving new ways to the improvement of patient care [81, 82].

### Genomics

Genomics, the first and most widely known -omics’ technology, studies the genetic profile of organisms either with respect to the whole genome or by targeting exonic coding regions or analyzing single nucleotide polymorphisms (SNPs) [83]. These high-throughput technologies have provided better insight into CT, especially with regard to epidemiological patterns among the various serovars, evolution of new variants due to mutations, monitoring sexual networks, and observation of persistence and reinfection rates. Several methods have been implemented for CT genotyping such as restriction fragment length polymorphism (RFLP), sequencing methods, DNA microarrays, and WGS [9].

RFLP involves the typing of the amplified *ompA* gene using restriction enzymes, where each genotype produces fragments of varying lengths that are electrophoretically identified. This technique is more focused on looking into the epidemiological patterns of various genovars of CT but fails to perform in infections with mixed genotypes and to identify single nucleotide changes and possesses low discriminatory power [9]. Various studies have utilized this technique in genotyping CT strains isolated from diverse populations [84–87].

Hybridization methods have been the superior technologies in detecting mixed CT infections and in epidemiological studies. Advanced types in use are reverse line blot hybridization, reverse dot blot hybridization, and microsphere suspension array hybridization. These techniques are based on hybridization of the amplified DNA with probes labeled on nylon membranes or carboxylated beads and results are noted either by blot formation or by analyzing the signals generated [9]. Studies conducted using these methods have highlighted their role in revealing multiple genotypes from a single clinical specimen and effective genotyping in large epidemiological studies [88–90]. However, poor resolution and inability to identify genovariants are major drawbacks [9].

The discriminatory power of CT typing has been increased with the introduction of high-resolution typing methods like MLVA and MLST [9, 10]. Several studies have shown their significance in determining genetic variants and in phylogenetic analyses [91–93]. But these expensive, equipment dependent methods are inefficient in diagnosing mixed infections leading to uninterpretable results. The emergence of DNA microarrays, as alternate technologies of genotyping requiring minimal sequencing, has been a major progress both in research and diagnostics. These high-resolution methods are rapid, less expensive with high sensitivity and specificity. Moreover, these methods can detect mixed infections making them relevant in clinical and epidemiological setups [94]. Studies have shown their superiority to MLST techniques in genotyping with regard to rapidity, relatively low cost and easier analysis of results. Also, they have proven to be comparable with these high-resolution methods in terms of sensitivity and specificity making them powerful alternative tools [95].

WGS has emerged as a vital tool for public health surveillance, molecular epidemiology of infectious diseases, and determination of antimicrobial drug resistance. This method is known to provide higher resolution and accuracy than the classical molecular typing methods. In CT diagnosis, this technology has been a promising solution in recognizing recurrent and persistent infections, thus helping to improve clinical management and disease control [96]. Clarity on the concept of reductive evolution involving genome degradation in CT has been enabled by WGS [97]. Though recombination has been previously considered uncertain in CT, owing to its intracellular nature, whole genome analysis has revealed the recombination machinery within the genome and the capability among clinical strains to recombine naturally or in cell cultures. Recombination, an inherent property responsible for diversity among species, was found to occur not only in strains with tropism to similar tissues but also between strains possessing tropism to different tissues. This correlated with the incidence of mixed and cross-site infections [98]. Chlamydia culture, a cumbersome and time-consuming technique, was previously a prerequisite for obtaining DNA for WGS, but the introduction of culture-independent methodologies has resolved these glitches making WGS popular as the ultimate typing tool [9, 10, 99]. The application of the technique directly on clinical specimens is very advantageous, as it can lead to a rapid diagnosis and thus accelerate the clinical response. WGS provides an insight into the epidemiological pattern and genetics of CT, which could eventually aid in tracing sources and investigating transmission networks [97, 99]. Borges V et al. studying the LGV outbreak among retro positive MSM performed WGS from anorectal samples. This helped them to notify the outbreak of LGV and the strain causing this outbreak was found to be unique (L2b/D-Da). This study highlights the role WGS can play in identifying and also characterizing LGV directly from clinical specimens [100].

### Transcriptomics

Even with genomic studies providing highly accurate data, the information generated is mostly static and not functional [83]. Advanced technologies in the -omics’ cascade have assisted in overcoming these barriers. Transcriptomics, the field analyzing the host mRNAs, have played a significant role in elucidation of the pathophysiological changes and varied gene expressions occurring in host cells during disease [101]. Analysis of host mRNAs provides a direct approach to cell and tissue-specific gene expressions which can be fundamental in apprehending the effect of altered transcriptome profiles during CT infections. Hayward RJ et al. have proposed the utility of single-cell approaches which could identify early infection biomarkers in host cells using single-cell RNA-Seq (sc RNA-Seq) and in understanding the intricate host responses during CT infections [102]. Detecting mRNA can be a potential marker for determining chlamydia viability indicative of true infections, which is unlikely using NAAT methodologies. Also, it can be used to determine the gene expressions at various stages of the chlamydia life cycle. A study by Zheng X et al. highlights the significance of the blood mRNA profile in diagnosing sexually transmitted infection-induced endometritis in women, which would further promote extensive screening for STIs in order to prevent further sequelae [103]. However, studies involving mRNAs as templates can be challenging due to their fragile nature [104], and the interpretation of data generated by these high-end technologies requires high expertise. Therefore, further research is necessary in order to validate these tests in the diagnostic setup.

### Proteomics

Proteomics, the qualitative or quantitative study of the proteome, which comprises the entire set of proteins of an organism, could help develop new diagnostic and prognostic biomarkers. Though transcriptional profiles provide insight into host–pathogen relations, post-transcriptional alterations could generate modified host proteins which can be effectively evaluated by proteomics [83]. Chlamydia, being an obligate intracellular pathogen, is known to intensely remodel the host proteome during infections for its replication and survival. This concept has been elucidated in a study conducted by Olive AJ et al. utilizing the global protein stability (GPS) platform, which demonstrates altered stability of host proteins and sequential manipulation of host pathways during CT infections. Analysis of these changes in host proteins could contribute to the understanding of the host–pathogen interactions and the development of therapeutic regimens [105]. Interferon-γ (IFN-γ), released as a mechanism of cell-mediated immune response during CT infections, disturbs the normal developmental cycle of the pathogen by decreasing the tryptophan synthesis, leading to the formation of aberrant reticulate bodies (ARBs) which are non-replicating but viable. Quantitative proteome analysis of these ARBs has shown increased levels of tryptophan synthase subunits, likely markers of persistence, which is a key feature leading to chronic infections and escape from immune responses [106]. Though ample data is produced by these proteomic tools, complexity in analysis can be a major limitation. Validating these technologies in the future can advance them as promising biomarkers in clinical diagnosis [79].

### Metabolomics 

Metabolomics, the most recent layer of the -omics’ cascade, focuses on the quantitative estimation of metabolites produced either during the host–pathogen interaction or during interventions. These can prove to be significant biomarkers indicative of infection, as well as in monitoring the effectiveness of therapy [101]. This field is found to be interlinked to the other -omics’ strata, as the levels of the metabolites produced are complementary to the variations in transcriptome and proteome [83]. Research studies conducted in order to demonstrate these compounds and tag them as indicators of infections have been on the rise in the field of chlamydia diagnosis. A study by Foschi C et al. involving a proton-based nuclear magnetic resonance spectroscopy has compared the urine metabolome of women with CT genito-urinary infection with that of CT negative women. An increased concentration of metabolites like sucrose, mannitol, lactate, and pyruvate were observed in CT-infected women, which were not found in CT negative women [107]. Also, in a study adapting a similar methodology for the metabolic profiling of vaginal swabs from CT-infected women and healthy women, specimens from CT-infected women exhibited altered nitrogen metabolism with decreased levels of amino acids and biogenic amines. Variations in these metabolites were proposed to be indicative of CT infection, thus implicating them as potential biomarkers [108]. However, further research is essential in order to be able to associate these low molecular weight compounds with the pathogenic mechanisms of CT and to comment on their diagnostic or prognostic value [107, 108]. Simplified data interpretation and cost-effectiveness could drive the utilization of these methods in clinical practice [101].

The advantages and disadvantages of the various -omics’ technologies discussed above are summarized in Table 2.

**Table 2:** Comparison of -omics’ technologies in CT diagnosis

-omics’ methodology	Advantages	Disadvantages	References
Genomics			
a) RFLP	• No requirement of chlamydia culture • Rapid, easy to perform technique • Used in epidemiological studies	• Presence of atypical restriction patterns • Low ability to detect mixed infections, genovariants • Analytically difficult	Rawre et al. [9] de Vries et al. [10] Gao et al. [84]
b) Hybridization methods (RLBH, RDBH, MSA)	• Ability to detect mixed infections • Utility in epidemiological studies	• Low resolution • Poor detection of genovariants	Rawre et al. [9] Huang et al. [88]
c) Sequencing methods (MLST, MLVA)	• High resolution • Detection of genovariants • Phylogenetic analysis	• Failure to detect mixed infections • Non-interpretable results • Expensive, labor-intensive	Rawre et al. [9] Herrmann et al. [91] Gravningen et al. [92] Peuchant et al. [93]
d) DNA microarrays	• Rapid • High resolution • Short turn-around-time • Detection of mixed infections • Easier analysis	• Expensive for routine use	Rawre et al. [9] Gallo Vaulet et al. [94] Christerson et al. [95]
e) Whole-genome sequencing	• High resolution, accuracy • Detection of mixed infections, genovariants • Generation of large data for understanding diversity, evolution, and antimicrobial resistance	• High cost • Expertise for interpretation • Longer turnaround time	Rawre et al. [9] Seth-Smith et al. [99] Christiansen et al. [109]
Transcriptomics	• Detection of altered gene expressions in infected cells • Detection of bacterial viability	• Expensive • Technically difficult • Significance as diagnostic tests to be validated	Hayward et al. [102] Zheng et al. [103] Janssen et al. [104]
Proteomics	• Detection of host–pathogen interactions • Rapid	• Significance as diagnostic tests to be validated	Olive et al. [105] Østergaard et al. [106]
Metabolomics	• Markers of active infection • Rapid	• Significance as diagnostic tests to be validated • Expensive	Foschi et al. [107] Parolin et al. [108]

*RFLP* restriction fragment length polymorphism, *RLBH* reverse line blot hybridization, *RDBH* reverse dot blot hybridization, *MSA* microsphere suspension array, *MLST* multi-locus sequence typing, *MLVA* multi-locus variable number tandem repeats analysis

### -omics’: an integrated approach

Considering the disadvantages associated with individual -omics’ methodologies, their implementation in clinical practice is not yet well established. In very few instances, these methodologies have been found to outpace the already available testing strategies [80]. With regard to the laboratory viewpoint, these technologies demand high-end equipment, technical expertise for analyzing the enormous data generated and associated high cost [101]. However, research studies utilizing these techniques have elucidated their role in diagnosis, even though they are not yet implemented in routine practice. Integrating the various layers of the -omics’ cascade enables a better understanding of the underlying factors in disease. In CT diagnosis, this integrated approach could play a pivotal role in unmasking the hidden patterns of this pathogen, deemed to be associated with the complications of CT infections. It could also aid in monitoring transmission networks and eventually disease prevention. Furthermore, awareness of the strengths and limitations of these high-throughput methodologies among clinicians is a prerequisite for their effective introduction into clinical practice.

## Conclusion

The accurate and cost-effective diagnosis of STIs, while keeping in mind that a syndromic approach is needed, poses a major challenge, particularly in developing and underdeveloped countries. Implementing highly sensitive and specific POC tests that are rapid, accessible, and affordable is a necessity in all settings. The diagnosis of CT genital infections has come a long way, from the less-sensitive culture techniques to the non-culture methods, to the advent of highly accurate molecular technologies. -omics’ technologies, especially when used in an integrated manner, can be very promising solutions. Better awareness of their diagnostic and prognostic value, supporting infrastructure and available research studies for further validation, is necessary for the success and widespread implementation of these technologies in CT diagnosis and management.
